# A monthly sub-national Harmonized Food Insecurity Dataset for comprehensive analysis and predictive modeling

**DOI:** 10.1038/s41597-025-05034-4

**Published:** 2025-05-05

**Authors:** Melissande Machefer, Michele Ronco, Anne-Claire Thomas, Michael Assouline, Melanie Rabier, Christina Corbane, Felix Rembold

**Affiliations:** 1 European Commission, Joint Research Centre (JRC), Ispra, 21027 Italy; 2 Action Against Hunger, Regional Office for West and Central Africa, Dakar, 29621 Senegal; 3 Previously at IPC, Global Support Unit, Rome, 00153 Italy; 4 Present Address: OCHA, Centre for Humanitarian Data, The Hague, 2511 CJ Netherlands

**Keywords:** Developing world, Environmental social sciences

## Abstract

Food insecurity is a complex, multidimensional concept challenging to measure comprehensively. Effective anticipation, monitoring, and mitigation of food crises require timely and comprehensive global data. This paper introduces the Harmonized Food Insecurity Dataset (HFID), an open-source resource consolidating four key data sources: the Integrated Food Security Phase Classification (IPC)/Cadre Harmonisé (CH) phases, the Famine Early Warning Systems Network (FEWS NET) IPC-compatible phases, and the World Food Program’s (WFP) Food Consumption Score (FCS) and reduced Coping Strategy Index (rCSI). Updated monthly and using a common reference system for administrative units, the HFID provides comprehensive spatial and temporal coverage to the extent permitted by the available data. It serves as a vital tool for food insecurity experts and humanitarian agencies, providing a unified resource for analyzing food insecurity conditions and highlighting global data disparities and gaps. The scientific community can also leverage the HFID to develop data-driven predictive models, enhancing the capacity to forecast and prevent future food crises.

## Background & Summary

Achieving the United Nations Sustainable Development Goals 2 – preventing food shortages and reducing global hunger – is an imperative endeavor^1^. A critical step toward this ambitious goal is the establishment of continuous and reliable monitoring of food insecurity situation, striving for the broadest geographic coverage and the highest temporal frequency possible. Anticipating and understanding the evolution of food insecurity is essential for effective policy-making. Despite the need for consistently updated data, especially in remote and often most vulnerable areas, this objective remains challenging. Significant efforts have been made by various institutions and networks towards providing the essential data needed to inform strategies that can preempt and alleviate food crises and with tools to visualise and monitor the situation. Notable among these are the campaigns and methods supported by the Cadre Harmonisé (CH), the Food and Agriculture Organization (FAO), the Famine Early Warning Systems Network (FEWS NET), the Integrated Food Security Phase Classification (IPC), the World Bank (WB), and the World Food Programme (WFP).

However, monitoring food insecurity at a global level faces significant challenges due to the uneven coverage of countries and regions. Moreover, disparate criteria for measuring food insecurity are employed, depending on the goals of the stakeholders. The complexity of food insecurity, influenced by context specific factors, challenges the development of a universal, objective measure, due to its inherent diversity and multi-dimensionality^2^. The frequency of data collection and the periods monitored of the various food insecurity indicators often vary, reflecting the differing needs of each initiative at local level. Additionally, geographical inconsistencies arise from the use of various reference systems for administrative boundaries and locations. Consequently, the current landscape of food insecurity assessment is marked by a lack of cohesion, highlighting the need for a more integrated and systematic methodology at a global level^3–7^. Data scientist practitioners have attempted to leverage publicly available datasets, proposing models for nowcasting and forecasting a stand alone target indicator^8–18^. Using multiple proxies and targets to assess food insecurity would be more effective than a single measure, as it yields a fuller, multidimensional understanding of food insecurity. However, some food insecurity datasets remain unused, due to recent availability, but also the complexity of the definition and of the technical direct access.

To bridge these gaps, we have developed the Harmonized Food Insecurity Dataset (HFID)^19^, a comprehensive compilation of major available sub-national food insecurity data sources with updates on a sub-annual basis. Our dataset spans from June 2007 to May 2024 and encompasses four key variables: the IPC/CH phases^20^, the FEWS NET-IPC compatible phases^21^ and prevalences of population with insufficient food consumption based on single indicators of food consumption: the Food Consumption Score (FCS) and reduced Coping Strategy Index (rCSI), both sourced from WFP^22^ through two different methods. Records within the dataset correspond to the first or second administrative level of the countries, harmonized using the Global Administrative Areas (GADM) database as the standard reference for the nomenclature of administrative units^23^. The HFID^19^ comprises 311, 838 records, encompassing 5, 508 units and 1, 264 units respectively corresponding to the second and first administrative division across 80 countries. By integrating these variables within a unified temporal and spatial framework, we enable a thorough and unparalleled comparative analysis of food insecurity trends derived from various datasets. This dataset can serve as a foundation for analyzing historical trends, drivers, and future projections in the food crisis landscape worldwide.

To effectively track and improve humanitarian aid and development funding, policymakers and donors require a comprehensive analysis of food insecurity trends over time and geography, leveraging data from all available sources. No current web-vizualisation (IPC (https://www.ipcinfo.org/), CH (https://www.cadreharmonise.org/), FEWS NET (https://fews.net/), HungerMap (https://hungermap.wfp.org/), PRISM (https://prism.dakar.wfp.org/), GAFS (https://www.gafs.info/home/) presents all food insecurity indices available and historical trends at sub-national level. The HFID^19^ fuels a one-stop shop for impacts decision making at sub-national level, with identified users such as aid programs managers, experts in the Global Report on Food Crisis, and IPC/CH and FEWSNET analysts. It offers a powerful solution, enabling the examination of historical trends and patterns, as well as ongoing monitoring of food insecurity through multi-partner analytical approaches and single outcome indicators, and retro-analysis of experts’ predictions. This serves as an essential tool for identifying where data is available and, importantly, where it is lacking. By highlighting these gaps, it encourages more focused data collection efforts in underrepresented regions or countries. Additionally, it helps identify areas experiencing persistent food crises, which may have a chronic component.

While the HFID^19^ provides an invaluable resource for analyzing food insecurity, its temporal discontinuity presents a notable limitation, as many datasets are only available for select periods of the full timeline. To overcome this, one could integrate proxy data or utilize related drivers of food insecurity, such as climate, economic, and conflict indicators, to fill in the gaps during periods where HFID variables are absent. Also developing an aggregated indicator by combining the various HFID variables through expert judgment or data-driven methods could provide a simplified measure of food insecurity. This approach would allow experts to determine the most suitable indicator to use in specific regions or periods, such as choosing between FCS and rCSI or between FEWSNET and IPC/CH, thus ensuring more consistent information is available even when some indicators are missing.

For modelers, the HFID^19^ offers a valuable, analysis-ready dataset that can also drive the development of advanced modeling techniques in the field of food insecurity. Notably, most existing studies have focused on predicting single indicators. There is still no comprehensive comparison of the factors driving FEWSNET and IPC/CH phases, presenting an opportunity to compare models for these indices to uncover underlying differences. There seems to be an absence of efforts to create forecasts that combine various food insecurity indices, utilizing either multi-output models or the construction of an aggregate target index^24^. To address this gap, it would be intriguing to enrich the HFID’s target variables with predictors that capture the recognized drivers of food crises, associating different food insecurity targets with the varying impacts of these predictors^25^. Methods such as causal forests or reinforcement learning could help identify causal relationships between different factors and food insecurity outcomes, which can be crucial for designing effective interventions. The temporally discontinuous food insecurity indices in the HFID^19^ can be further enhanced through data completion modeling, which can effectively utilize the added continuous predictors. Moreover, these added predictors can provide valuable insights into the underlying mechanisms and dimensions of food insecurity, revealing a more nuanced understanding of the complex relationships at play. Clustering techniques could also be employed to identify distinct regional patterns. By exploring these applications, we anticipate a significant advancement in our comprehension of food insecurity, including the intricate interactions between factors such as weather, climate, conflict, and other disruptive events.

## Methods

Several food insecurity raw data sources have been used to build the HFID^19^. We collected the IPC/CH and FEWS NET-IPC compatible current situation phases through the freely accessible APIs^26,27^, respectively referred hereafter with phase-IPC/CH and phase-FEWS. The prevalence of population with insufficient food consumption based on single indicators FCS and rCSI, were instead retrieved from two different sources: a publicly available historical dataset^28^ from the Literature (LIT) covering 2006-2021, called in the following WFP-LIT, and a dedicated data dump with all historical records from Real Time (RT) monitoring also available (only last 500 days) in the HungerMap^29^, called in the following WFP-RT. We hereafter refer to the population prevalence of insufficient FCS - both poor and borderline - and rCSI of 19 or above, as FCS-LIT and rCSI-LIT for data from WFP-LIT, and as FCS-RT and rCSI-RT for data from WFP-RT, indicating the respective data sources for these indicators. These thresholds used for computing population prevalence of insufficient food consumption for FCS and rCSI indicators are the ones used for defining IPC/CH Phase 3 and higher (see the IPC Acute Food Insecurity Reference Table^30^).

Both FEWS NET and IPC/CH provide a unified classification of Acute Food Insecurity (AFI) of a geographic area in the form of five classes: Phase 1 is None/Minimal Food Insecurity, Phase 2 is Stressed, Phase 3 is Crisis, Phase 4 is Emergency, and Phase 5 is Catastrophe/Famine. The “overall_phase” key corresponds to phase from 1 to 6, with numbers from 1 to 4 matching their corresponding IPC/ CH Phase, while Phase 5 that can take either the value 5 (Famine with solid evidence) or the value 6 represents Famine (with reasonable evidence), see also the API documentation (https://observablehq.com/@ipc/ipc-api-extended-documentation). To associate a phase to a geographical area, the analysts must use all relevant and reliable evidence on food insecurity for that area and period of analysis. The evidence used results from a combination of outcomes indicators (such as FCS) and other contributing factors (such as the presence of conflicts). Such a framework identifies food consumption, livelihood change as first order outcome of food insecurity and nutritional status, and, mortality as second order outcome indicators. The analysis protocols require specific proxy indicators on food consumption and livelihood change to be included. The Reference Table contains all the indicators considered as direct evidence and provides comparable cut-offs associated with the five IPC phases for each indicator^30^. The FEWS NET analyses are IPC-compatible, as they utilize the same protocols, but are not always based on a multi-partner consensus. This scenario may occur when specific organizations request analyses and classifications of food insecurity conditions without a collaborative agreement among technical experts. For example, this might be due to the pressing need for immediate action in response to an urgent food insecurity situation.

The FCS and rCSI are food indicators widely collected and used by WFP and partners and which are referred in the Reference Table to document the food consumption outcome^30^. The FCS intends to measure the quality and quantity of household’s food access, however only its association with energy food consumption has been validated^31^. This score aggregates household-level data on dietary diversity, frequency of food consumption and relative nutritional value of food consumed over the last 7 days^32^. Based on the value obtained, a household’s food consumption is further classified into three categories: poor, borderline, or acceptable. The WFP-LIT and RT-LIT data sources provide the sum of the prevalence of households with poor and borderline conditions which is called insufficient food consumption. The rCSI measures the frequency and severity of behaviors in which people have engaged in the last 7 days in response to challenges in accessing food^6^. It aggregates household data on the use of five pre-weighted coping strategies in the last 7 days. These data sources provide the prevalence of households with an rCSI score above 19^14^. Both food consumption indicators are collected with household surveys carried out by national institutions, or international organizations. The data collected in these surveys are not always openly accessible. The WFP provides daily estimations of the prevalence insufficient food consumption and crisis or above coping strategies obtained by phone surveys (the so-called mobile Vulnerability Analysis and Monitoring, mVAM) combined with in-person surveying through instruments like the Comprehensive Food Security and Vulnerability Analysis (CFSVA) and the Emergency Food Security Assessment (EFSA). Previous studies have compiled several years of WFP population prevalence of insufficient food consumption indicators at ADMIN1 level^14,16^. In the HFID^19^, from the WFP-LIT source, we only include the face to face records as they are the only ones validated. The WFP-RT only contains phone surveys records (mVAM), collected using the remote modality, hence the separation of the variables in the HFID (FCS-LIT, rCSI-LIT and FCS-RT, rCSI-RT).

The GADM^23^ dataset, composed of worldwide information on administrative boundaries at country and lower levels of sub-division, was used to have a common standardised reference for sub-national administrative units worldwide. In particular, we aimed at harmonizing the above mentioned food insecurity raw data sources into a dataset of monthly entries at GADM administrative level 2.

All raw sources contain information on the location of the records in the form of (1) shape files with geometries or (2) with administrative name of each area concerned. However different reference units are used (e.g. livelihood zones in the case of FEWS NET analyses). To harmonise geographies, we intersected the original geometries with the GADM areas at either ADMIN1 (for WFP population prevalences) or ADMIN2 granularity (for FEWS NET and IPC/CH analyses). Then each record was assigned to the GADM area with the largest intersection, provided that the sum of all intersections was covering more than 10% of the original area. In case of geometries from raw sources with smaller units of analysis than the GADM ADMIN2, a potential artefact could be expected from this spatial-based approximation approach. On this point, special attention is drawn on Haiti and Somalia from IPC/CH source and countries including very small livelihood zones in from FEWSNET source. Population prevalences were then calculated again for all subdivisions within a given larger region. For WFP-LIT, due to the lack of geo-referenced metadata, a pre-processing step to obtain geometries from administrative names was necessary to implement by using GPT4.0 together with Open Street Map. Geometries were assigned to the original locations by using *osmnx* (a python package based on Open Street Map) and, when it was not possible to identify a given unit, we first corrected any misspelling or uncommon names by using the OpenAI API for GPT4.0.

In order to build monthly entries, IPC/CH phases were repeated each month during the validity in the analysis, while FEWS NET phases, available three to four times per year, were only considered valid for the reference month. For the daily available WFP-RT raw indicators, we computed several monthly statistics – minimum, maximum, average – and for the daily available WFP-LIT raw indicators we computed the monthly average.

Whenever available, we also include information on distributed humanitarian aid as a boolean variable when required for analyses. The presence of refugees and settlements is recorded as a boolean when indicated in the IPC API, following the IPC mapping protocol (see IPC Manual^30^, page 78). It is important to note that displaced populations might be included in the IPC’s analysis even if they are not visually represented or classified as separate units, and therefore may not be listed as such in the API.

**Fig. 1:**
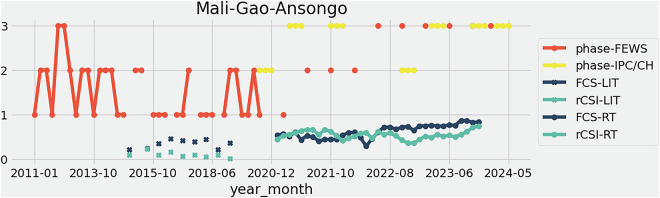
This figure shows time series of available variables values in the HFID (phase-FEWS, phase-IPC/CH, FCS-LIT, rCSI-LIT, FCS-RT, rCSI-RT) for the sub-region of Ansongo, Gao, Mali. A unique y-axis is used for display, reminding the reader that those variables represent different quantities.

**Fig. 2:**
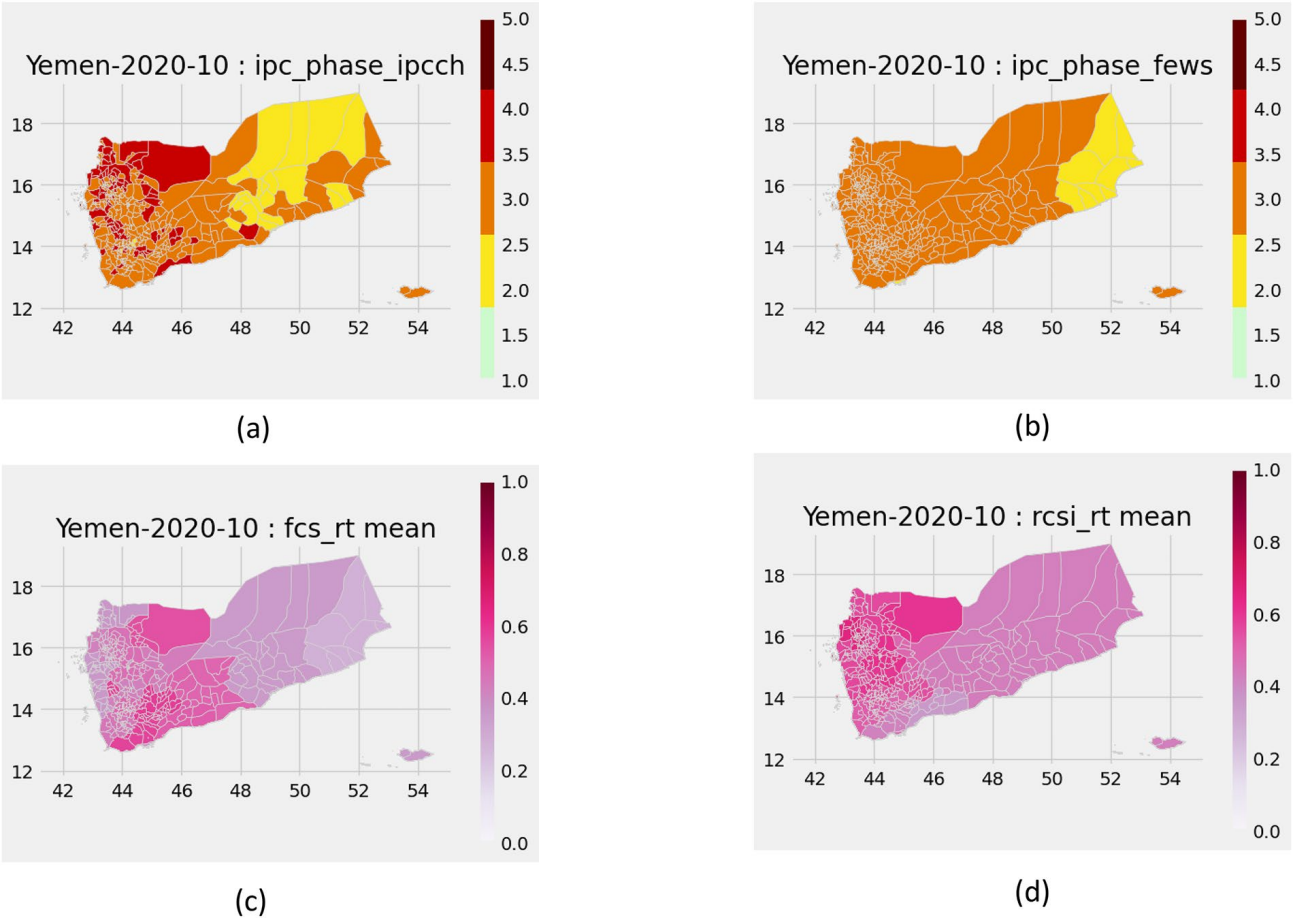
An example of four mapped variables of the HFID dataset for Yemen in October 2020: (**a**) phase-IPC/CH (variable *ipc_phase_ipcch*), (**b**) phase-FEWS (variable *ipc_phase_fews*), (**d**) monthly average of FCS-RT (variable *fcs_rt mean*), (**e**) monthly average of rCSI-RT (variable *rcsi_rt mean*).

Finally, we merge all separate sources into a unique set of available datepoints at ADMIN2 and/or ADMIN1 (when no ADMIN2 data is available, i.e. in countries where only WFP population prevalence are found) entries. In the final dataset, WFP data are thus repeated for all those ADMIN2 units present in a given ADMIN1 when we have also records from IPC/CH or FEWS NET. An illustration of several variables timeseries of the HFID plotted for the ADMIN2 of Ansongo, located in Gao, Mali, is found in Fig. 1. An example of four HFID indices for Yemen on October 2020 is given in Fig. 2.

## Data Records

The HFID^19^ is available on 10.5281/zenodo.15017473. The dataset consists of files with administrative units geometries and one file with HFID variables. The geometries are found (1) in the GADM geometries with distinct administrative levels 0,1 and 2 (GADM.zip), (2) in the geopackage file with combined geometries at relevant administrative level with exact geometries (hfid_all_geom.gpkg) or simplified geometries (simplified_hfid_geom.gpkg). The GADM.zip file contains the shapefiles with administrative level 0 (gadm_410_L0), level 1 (gadm_410_L1) and administrative level 2 (gadm_410_L2) reference geometries.

The data table with the HFID variables (HFID_hv1.csv) is composed of several columns:

- *year_month*: indicates the year and month of the record in the format “%Y-%m”.
- *ADMIN_0*: name of the administrative level 0 (country) as defined in the GADM.
- *ADMIN_1*: name of the administrative level 1 (region) as defined in the GADM.
- *ADMIN_2*: name of the administrative level 2 (sub-region) as defined in the GADM.
- *ipc_phase_fews*: FEWS NET-IPC compatible phase classification (phase-FEWS variable) if available, else NaN. Possible values: 1,2,3,4,5.
- *ha_fews*: 1 if humanitarian aid has been distributed (owing to FEWS NET source), else NaN.
- *ipc_phase_ipcch*: IPC/CH Phase classification (phase-IPC/CH variable) if available, else NaN. Possible values: 1,2,3,4,5,6.
- *ha_ipcch*: 1 if humanitarian aid has been distributed (owing to IPC API source), else NaN.
- *set_ipcch*: 1 if HouseHold Group or Internally Displaced People or Urban settlements are found in the area (owing to IPC API source), else NaN.
- *rfg_ipcch*: 1 if refugees are found in the area (owing to IPC API source), else NaN.
- *fcs_lit*: monthly average of population prevalence of insufficient food consumption score from the WFP-LIT source (FCS-LIT variable) if available, else NaN. Possible values: between 0 and 1.
- *rcsi_lit* monthly average of population prevalence of crisis or above reduced Coping Strategy Index from the WFP-LIT source (rCSI-LIT variable) if available, else NaN. Possible values: between 0 and 1.
- *fcs_rt mean*: monthly average of population prevalence of insufficient food consumption score from the WFP-RT source (FCS-RT variable) if available, else NaN. Possible values: between 0 and 1.
- *fcs_rt max*: monthly maximum of population prevalence of insufficient food consumption score from the WFP-RT sourceif available, else NaN. Possible values: between 0 and 1.
- *fcs_rt min*: monthly minimum of population prevalence of insufficient food consumption score from the WFP-RT source if available, else NaN. Possible values: between 0 and 1.
- *rcsi_rt mean*: monthly average of population prevalence of crisis or above reduced Coping Strategy Index from the WFP-RT source (rCSI-RT variable) if available, else NaN. Possible values: between 0 and 1.
- *rcsi_rt max*: monthly maximum of population prevalence of crisis or above reduced Coping Strategy Index from the WFP-RT source if available, else NaN. Possible values: between 0 and 1.
- *rcsi_rt min*: monthly minimum of population prevalence of crisis or above reduced Coping Strategy Index from the WFP-RT source if available, else NaN. Possible values: between 0 and 1.
- *iso2*: ISO 3166-1 code for country in two letters.
- *iso3*: ISO 3166-1 code for country in three letters.
- *region*: region name from the United Nations Geoscheme.

## Technical Validation

One of the primary objectives of the HFID^19^is to facilitate direct comparisons, at a single place, of various food insecurity variables across different areas and time periods, highlighting data availability and gaps. This is crucial for gaining a comprehensive understanding of regional conditions and enhancing the comparability of adopted food insecurity metrics. Besides IPC/CH phases, FEWS NET IPC-compatible phases, and WFP FCS and rCSI in HFID^19^, other datasets provide information on AFI. Notably, FAO’s Data in Emergencies (DIEM) offers prevalence of population derived from survey answers to individual questions used to collect single food security indicators (e.g., FCS, rCSI, HDDS, FIES) for 25 countries, aggregated at the sub-national level and updated sub-annually (typically twice a year) since 2021. However, the computation of single indicators from this data remains to be done before they can be effectively used, preventing for suitable inclusion in the HFID^19^. Additionally, the Living Standards Measurement Study - Integrated Surveys on Agriculture (LSMS-ISA) provides single indicators (e.g., FCS, rCSI, HDDS) for only 8 countries, and seldomly collected every 2-5 years since 2008, lacking spatio-temporal relevance for inclusion in the HFID^19^.

**Fig. 3:**
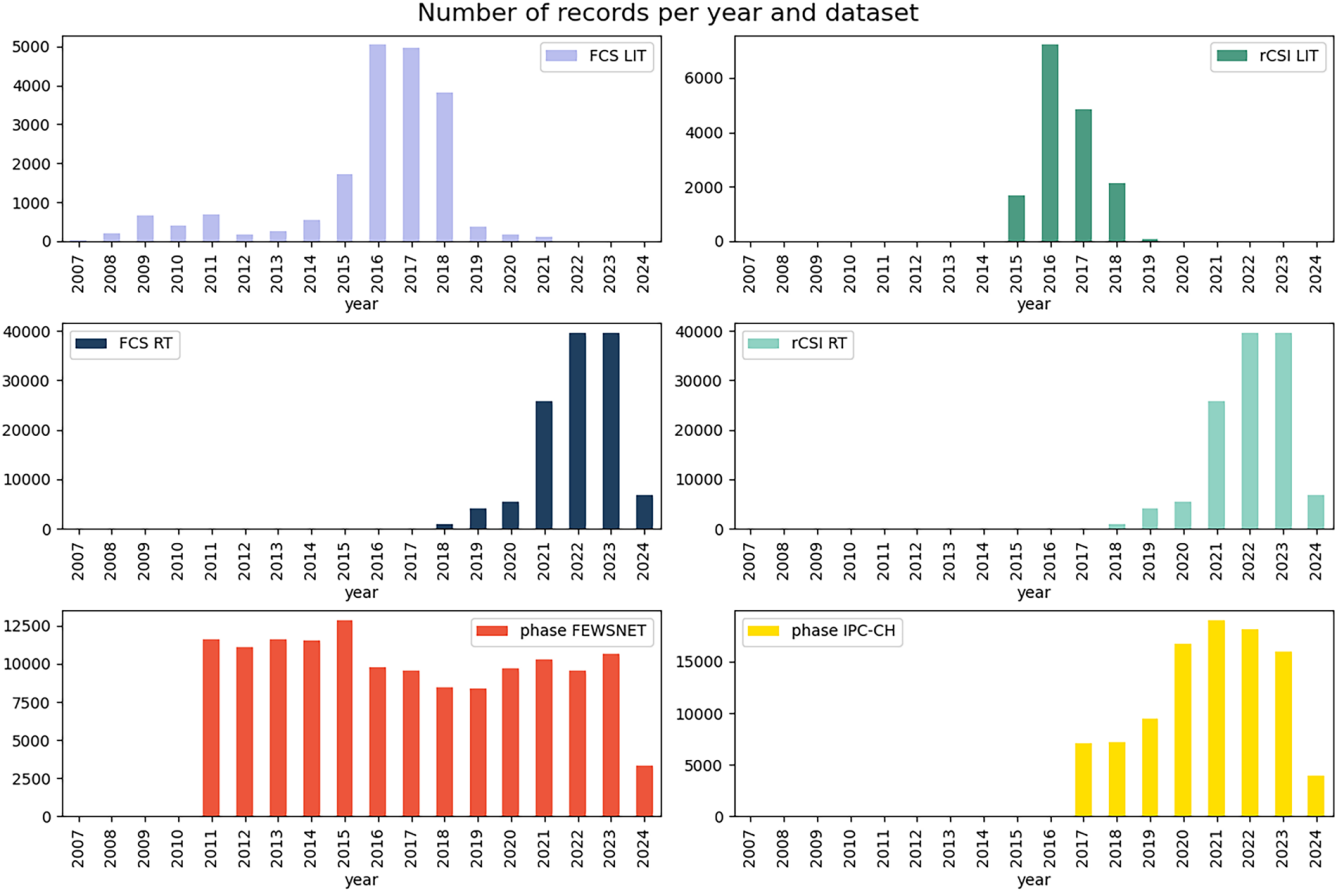
Number of records per year and variable of the HFID.

From Tables 1 and 2, we find that the HFID has the broadest geographical and historical coverage for the phase-IPC/CH and FCS-LIT variables, with around 100, 000 records in respectively 49 and 70 countries over 89 and 106 year-month entries and a high number of ADMIN2 covered. However, we highlight that FCS-LIT variable is only available until July 2021. We therefore subsequently analyse the temporal availability of the different variables of the HFID in Fig. 3, plotting the number of records per year. This presentation facilitates the understanding of data gaps. We observe that the WFP-LIT provides a peak of records in 2016, and other still ongoing sources (WFP-RT, phase-FEWS and phase-IPC/CH) offer quite stable number of records since 2022 at least (2024 is still ongoing year, thus with less records). We observe that for WFP-LIT, pre-2015 records are very sparse. The variable phase-FEWS provides the longest time serie and most steady and numerous records (since 2011, over 10, 000 yearly records), and the variable phase-IPC/CH has been ramping up from around 7, 000 records in 2017 up to more than 15, 000 since 2020.

**Table 1:** Number of unique countries (ADMIN0), and administrative level 1 (ADMIN1) and 2 (ADMIN2) areas, year-month entries for each variable of the HFID.

	ADMIN0	ADMIN1	ADMIN2	year-month
**FCS-RT**	33	494	3301	67
**rCSI-RT**	33	494	3301	67
**FCS-LIT**	**70**	**978**	3680	**106**
**rCSI-LIT**	*11*	*143*	*1434*	*46*
**phase-FEWS**	28	483	3911	*46*
**phase-IPC/CH**	**49**	678	**5022**	89

Formatting shows importance: **bold** = high, *italic* = low.

**Table 2:** Number of matching records between variables of the HFID. Note that mirrored pairs (repeated values) are not shown.

	FCS-RT	rCSI-RT	FCS-LIT	rCSI-LIT	phase-FEWS	phase-IPC/CH
**FCS-RT**	**122112**					
**rCSI-RT**	**122112**	**122112**				
**FCS-LIT**	*0*	*0*	19078			
**rCSI-LIT**	*0*	*0*	12583	15961		
**phase-FEWS**	23662	23662	3443	3666	**138334**	
**phase-IPC/CH**	32433	32433	*719*	*17*	12618	97478

Formatting indicates record volume: **bold** = high, normal = medium, *italic* = low.

**Fig. 4:**
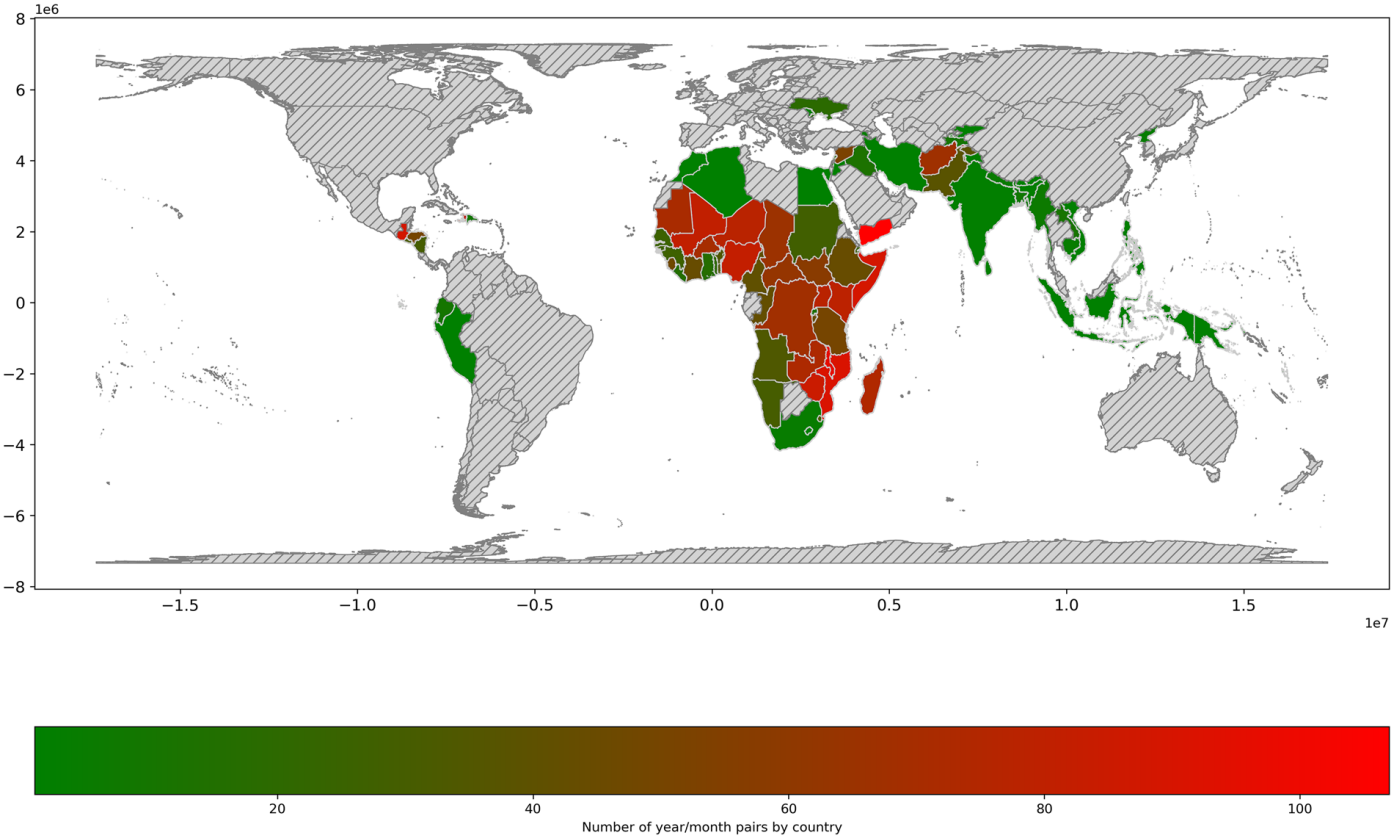
The world map coverage of HFID in terms of unique *year_month* occurrences by ADMIN0 (country) for all variables.

**Fig. 5:**
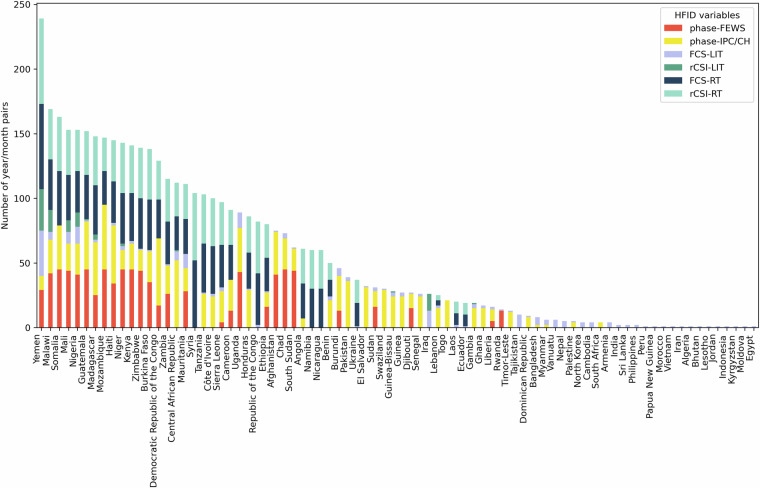
Number of unique year/month pairs by variable of the HFID and by ADMIN0 (country).

We also analyse the number of unique *year_month* occurrences by country (ADMIN0) in Fig. 4. The detail by HFID variable and country is provided in Fig. 5. More data both in terms of years and number of data sources is available for Latin America and the Caribbean, Western Africa, Middle Africa, Eastern Africa, Western Asia, and Southern Asia. On the contrary Eastern Europe, Central Asia, Eastern Asia, South-Eastern Asia and Oceania have very limited coverage in terms of the number of HFID variables as well as number of years. These illustrations also reveal that Yemen, the Sahelian countries Mali, Niger, Nigeria as well as Somalia, Malawi, Madagascar and Mozambique in Eastern Africa have the largest number of unique *year_month* occurrences.

**Fig. 6:**
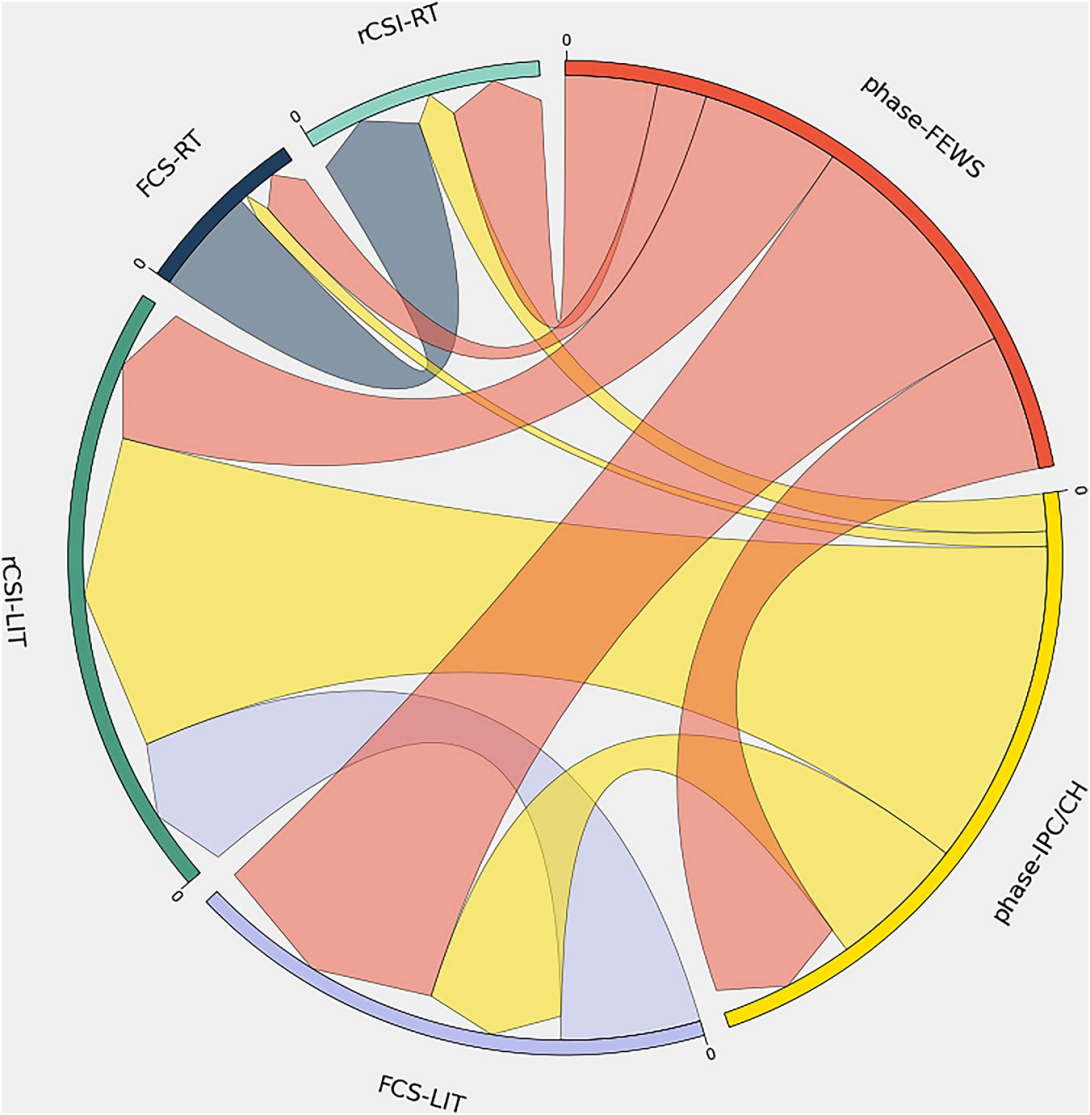
Chord plot showing the relation among all the variables in the HFID. The size of the arrows is proportional to the mutual information score among each pair of indicators. This provides an indication on the amount of information gained about one indicator through the observation of a second indicator in the HFID.

In order to compare values of the variables of the dataset, a basic statistical analysis consists in computing the correlation between variables, as found in Table 3. Some degree of correlation might exist due to the definition of some of the variables of the HFID, although not necessarily, noteworthy the different nature of the variables do not measure the same aspects of the dimension of food insecurity. We find that the FCS-LIT strongly correlates with all the other variables, with weakest correlation obtained for phase-IPC/CH which is also the variable for which there is however the least matching records (see column FCS-LIT in Table 2). Conversely, the phase-IPC/CH variable very weakly correlate with the FCS-RT while there is for a reasonable amount of records having both variables (32, 433 see Table 2). Weak correlation is also found for WFP-RT variables with phase-FEWS and phase-IPC/CH variables. Strong correlation is observed between phase-FEWS and phase-IPC/CH variables. Dependencies can also be non-linear and, thus, we also provide a comparison among all HFID indices in terms of mutual information score in Fig. 6. Mutual information is a way to measure how much one piece of data tells us about another. It captures both linear and nonlinear dependencies between variables, surpassing linear correlation measures in identifying complex relationships^33^. To visually represent this, we can utilize arrows: larger arrows indicate stronger connections, and it is important to note that the directions are arbitrary and can be interpreted in both ways. According to Fig. 6, WFP-LIT variables carry more information on the phases than WFP-RT data.

**Table 3:** Correlation of HFID variables with asterik (*) ratings indicating relative importance. Note that correlations between identical indicators (perfect correlation of 1) and mirrored pairs (repeated values) are not shown.

	FCS-RT	rCSI-RT	FCS-LIT	rCSI-LIT	phase-FEWS
**rCSI-RT**	0.39				
**rCSI-LIT**			0.65**		
**phase-FEWS**	0.32	0.49*	0.73**	0.45*	
**phase-IPC/CH**	0.02	0.30	0.53*	0.39	0.61**

**Fig. 7:**
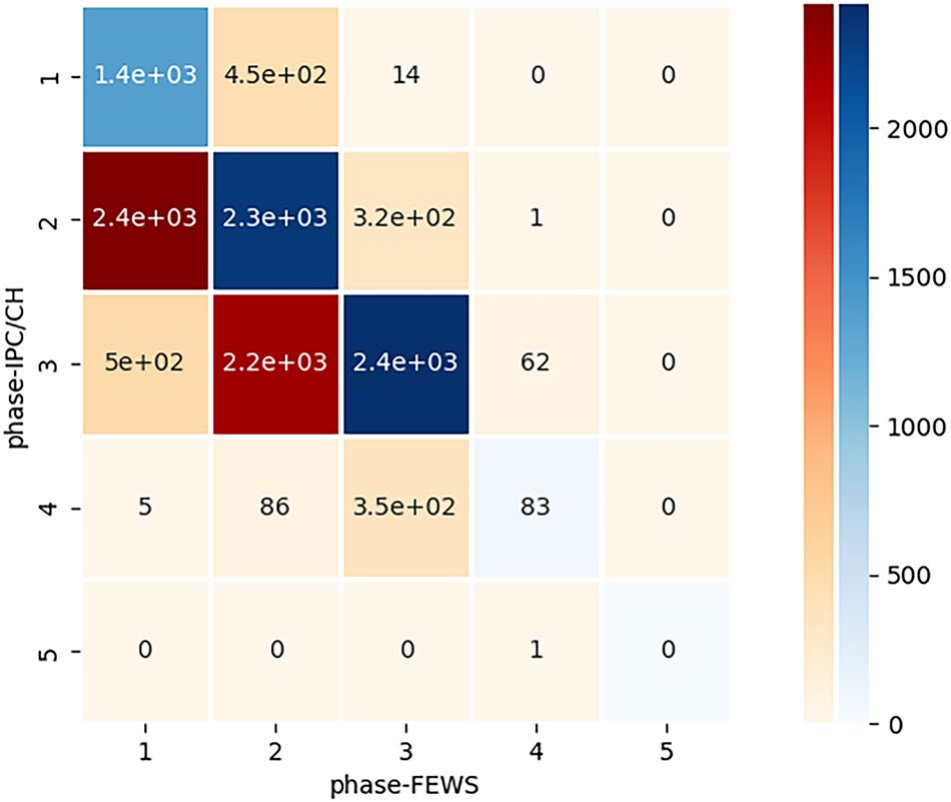
The figure illustrates the number of records in agreement between FEWS NET phases (phase-FEWS) in x-axis and IPC/CH phases (phase-IPC/CH) in y-axis. In an ideal scenario of complete agreement between the two classification schemes, the heatmap would form a diagonal matrix. Off-diagonal elements provide insight into areas of disagreement between the two schemes.

We compare the phase-FEWS and phase-IPC/CH values in Fig. 7. The HFID^19^ contains 12, 618 records with both sources, covering 22 countries. It is worth noting that while there is some general consensus between the two classification values, phase-FEWS consistently trend lower than phase-IPC/CH. Specifically, when the phase-IPC/CH is equal to 2, the phase-FEWS is observed to be either 1 or 2 with the same frequency. The same pattern is observed for phase-IPC equal to 3. As far as we know, this is the first comprehensive comparison of these two classification schemes.

**Fig. 8:**
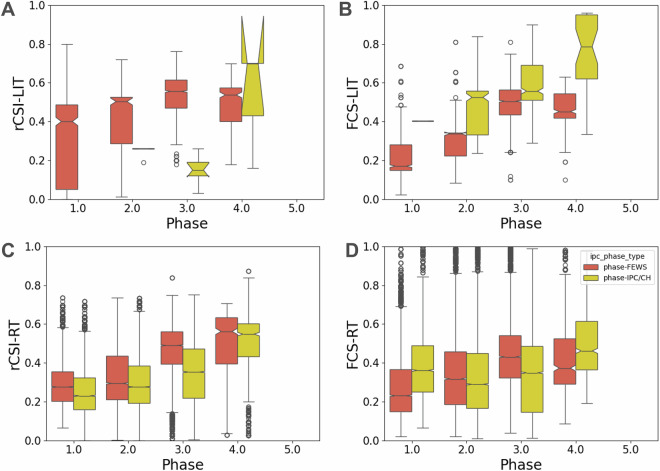
Box plots representing the distribution population prevalence of insufficient food consumption indicators (FCS, rCSI) from WFP-LIT and from WFP-RT, segmented into five different classes, distinguishing between FEWS NET and IPC/CH classifications.

In Fig. 8 we present a comparison of the FCS-LIT, FCS-RT, rCSI-LIT and rCSI-RT (for WFP-RT source, we take the monthly averaged variables *fcs_rt mean* and *fcs_rt mean*, see also the section Data Records) over all administrative units, both among themselves and in relation to both phase-IPC/CH and phase-FEWS variables, when spatio-temporal matching records are found. We show the results for the five phases provided by either FEWS NET(red boxes) or IPC/CH (yellow boxes). The number of records varies depending on the availability of pairs of indices for the same unit and year/month pair. As expected, we observe an increase in population prevalence of insufficient FCS and crisis or above rCSI across all phases. However, there are also differences between WFP-LIT and WFP-RT sources. Both rCSI-LIT (3, 666 events) and FCS-LIT (3, 443 events) saturate for crisis levels of phase-FEWS (i.e. ≥3), while the (median) prevalence keeps increasing almost linearly over phase-IPC/CH (take into account the very limited statistics, having only 17 events for rCSI-LIT and 719 for FCS-LIT). Conversely, WFP-RT data exhibits a more uniform pattern, indicating a similar trend for both classification schemes. Specifically, the rCSI-RT consistently increases for higher phase-FEWS (23, 662 events) and phase-IPC/CH (32, 433 events) classes, as shown in the bottom panel. FCS-RT also shows an increase in correlation with phase-FEWS (23, 662 events) and phase-IPC/CH (32, 433 events) values, but it is not as responsive to changes in phase (see also Table 3). This also indicates that rCSI-RT may be a more accurate predictor than FCS-RT for both FEWS NET and IPC/CH phase variables. However, it should be kept in mind that phase-IPC/CH 4 or more, FCS and rCSI are non defining characteristics in the IPC/CH protocol (see the IPC Acute Food Insecurity Reference Table^30^, page 37), meaning that analysts need to use other indicators (such as the Household Hunger Scale or Household Economy Scale) to discriminate between phase 4 and 5.

## Usage Notes

The code^19^ is reproducible and modulable in order to add future records, on a monthly or yearly basis, from existing sources and potential other sources of interest, like the population prevalence of answers to surveys enabling single food insecurity indicator computation from the FAO Data in Emergencies. The HFID^19^, structured in a tabular format, facilitates its integration into data-driven and machine learning analyses. Researchers and analysts can conveniently aggregate selected covariates at various levels of granularity, ranging from administrative level 2 to country level, suiting the specific needs of their studies. This adaptability makes the HFID^19^ an instrumental dataset for advanced analytical approaches in food insecurity.

We also provide with some functions automatically reading and processing the HFID^19^, in module *targets.utils.utils.py*:

- the function *read_hfid()* reads the HFID tabular dataset (HFID_hv1.csv) enabling the consideration that the “iso2” column with the “NA” value represents “Namibia” country and not a NaN.
- the function *read_admins()* reads all the shapefiles with specified administrative level reference geometries (GADM.zip). The processing includes the suppression of specific sub regions where inter-national conflicts of identity exist (e.g. China, India, Pakistan, Kosovo).
- the function *sanitize_high_phases()* allows to decide on the treatment to apply to IPC/CH Phase 6 (keep as such, remove it, or cap it to Phase 5).
- the function *add_admins2_geometry_to_hfid()* which combines the HFID tabular data and the administrative level geometries.

Finally, we propose some vizualisation functions, enabling to plot maps of the variables of the HFID^19^, in module *targets.utils.plot_utils.py*:

- the function *plot_phases_map()* plots a map with phases from FEWS NET or IPC/CH (variables phase-FEWS, phase-IPC/CH) at a certain date (*year_month*), respecting the IPC color code. An illustration of the output of this function is found in Fig. 2a,b.
- the function *plot_outcomes_indc_map()* plots a map with values from population prevalence of insufficient food consumption indicators from WFP-LIT and WFP-RT (variables FCS-LIT, rCSI-LIT, FCS-RT, rCSI-RT) at a certain date (*year_month*). An illustration of the output of this function is found in Fig. 2c,d.

## Data Availability

The code and relevant documentation are available on 10.5281/zenodo.15017473and are encouraged to be used citing our work in publications and reports, and using our license in further published codebase by external users.
